# Phenolic Profiling and Biological Potential of *Ficus curtipes* Corner Leaves and Stem Bark: 5-Lipoxygenase Inhibition and Interference with NO Levels in LPS-Stimulated RAW 264.7 Macrophages

**DOI:** 10.3390/biom9090400

**Published:** 2019-08-22

**Authors:** Catarina Andrade, Federico Ferreres, Nelson G. M. Gomes, Sutsawat Duangsrisai, Nattawut Srisombat, Srunya Vajrodaya, David M. Pereira, Angel Gil-Izquierdo, Paula B. Andrade, Patrícia Valentão

**Affiliations:** 1 REQUIMTE/LAQV, Laboratório de Farmacognosia, Departamento de Química, Faculdade de Farmácia, Universidade do Porto, R. Jorge Viterbo Ferreira, n° 228, 4050-313 Porto, Portugal; 2 Research Group on Quality, Safety and Bioactivity of Plant Foods, Department of Food Science and Technology, CEBAS (CSIC), P.O. Box 164, 30100 Murcia, Spain; 3 Department of Botany, Faculty of Science, Kasetsart University, Ngam Wong Wan Road, Chatuchak, Bangkok 10900, Thailand; 4 Center for Advanced Studies in Tropical Natural Resources, NRU-KU, Kasetsart University, Chatuchak, Bangkok 10900, Thailand

**Keywords:** aviculin, catechin, chlorogenic acid, cinchonain, procyanidin, vicenin-2, vitexin

## Abstract

The economic value of fig trees has been globally acknowledged due to their utilization in the food industry, being also frequently used in traditional medicine. While ubiquitously distributed in Southeast Asia, *Ficus curtipes* Corner remains uninvestigated concerning its biological properties and chemical profile. HPLC-DAD-ESI/MS*^n^* characterization of methanol extracts obtained from the stem bark and leaves allowed the identification and quantitation of 21 phenolic compounds for the first time; the stem bark was predominantly rich in flavan-3-ols and apigenin derivatives, while solely apigenin-di-glycosides have been identified and quantitated on the leaf extract. Both extracts inhibited 5-lipoxygenase (5-LOX) activity in a concentration-dependent manner, the one obtained from the stem bark being significantly more active (IC_50_ = 10.75 μg/mL). The effect of both extracts on lipopolysaccharide (LPS)-stimulated RAW 264.7 macrophages was evaluated, and while the stem bark extract did not lead to a noticeable interference on nitric oxide (NO) levels, the extract obtained from the leaves notably decreased NO and L-citrulline levels at concentrations ranging from 250 to 500 μg/mL. Herein, *F. curtipes* is valorized due to its modulatory effects on inflammatory mediators and also as a source of bioactive phenols, which may fuel further studies on the development of nutraceuticals.

## 1. Introduction

Over the past few years, research on multi-target molecules, particularly those obtained from natural sources, has been gaining ground on the treatment and/or prevention of several multi-factorial diseases [1]. In fact, approximately 50% of the approved drugs in the past 30 years were derived either directly or indirectly from natural products, predominantly from medicinal plants, playing a unique role as sources of both simple and complex bioactives [2]. Systematic studies based on the ethnomedicinal use of some plant species represent a useful approach for the elucidation of their therapeutic potential, both at a species or higher taxonomic levels [3]. However, and despite the multiple reports on the use of plants in traditional medicine, most species still lack scientific data on their biological properties and chemical profiles.

With a global distribution of approximately 800 species [4], the therapeutic and economic value of *Ficus* genus has been gaining increased attention, scientific evidence frequently supporting its therapeutic potential and allowing the isolation of biologically active compounds [5]. *Ficus carica* L., *Ficus religiosa* L., and *Ficus racemosa* L. have been the most widely investigated species, including several reports on their anti-inflammatory and antioxidant activities [6,7,8,9,10]. Worth highlighting is a study by *Park* et al. [6], demonstrating not only the strong antioxidant properties of an ethanol extract obtained from *F. carica* branches, but also significant anti-inflammatory-like effects in RAW 264.7 macrophages, reducing both nitric oxide (NO) and tumor necrosis factor-α (TNF-α) levels. A significant anti-inflammatory effect was also observed in mouse microglial BV-12 cells challenged with lipopolysaccharide (LPS) upon exposure to a methanol extract obtained from the leaves of *F. religiosa*, leading to the reduction of NO levels and pro-inflammatory cytokines [8]. The anti-inflammatory properties of *F. racemosa* was also demonstrated in in vivo studies carried out with a petroleum ether extract from *F. racemosa* leaves demonstrated a potent effect against acute pedal edema comparable to phenylbutazone, a prototype of a non-steroidal anti-inflammatory agent [10].

Unlike the abovementioned species, *Ficus curtipes* Corner remains to be investigated, particularly concerning its potential pharmacological effects. The passively pollinated monoecious fig tree is commonly found in India, Indonesia, Malaysia, Myanmar, Nepal, Thailand, and Vietnam [11], but despite its widespread distribution among the Asian continent, there are no records on its chemical profile and pharmacological effects. Prompted by previous reports on *Ficus* species, and considering the absence of data on the biological properties of *F. curtipes*, we found relevant to investigate the effect of methanol extracts obtained from the stem bark and leaves towards NO levels on LPS-stimulated RAW 264.7 macrophages, interference with the activity of 5-lipoxygenase (5-LOX) being assessed as well. As there are no reports on the bioactives’ content of the species, we also aimed to generate data on the polyphenolic composition of the plant through HPLC-DAD-ESI/MS*^n^*.

## 2. Materials and Methods

### 2.1. General Chemicals and Standards

*N*-(1-Naphthyl) ethylene-diamine dihydrochloride, methanol and acetonitrile were from Merck. 5-LOX from *Glycine max* (Type V; EC 1.13.11.12), linoleic acid, diacetyl monoxime, antipyrine, quercetin, sulphanilamide, sodium phosphate, phosphoric acid, thiazolyl blue tetrazolium bromide (MTT), LPS from *Escherichia coli*, *N*-[naphth-1-yl]ethylenediamine dihydrochloride, dimethyl sulfoxide (DMSO), l-arginine were purchased from Sigma-Aldrich (St. Louis, MO, USA). Sodium nitroprusside dihydrate (SNP) was acquired from Riedel-de Haën (St. Louis, MO, USA). The water was treated in a Milli-Q water purification system (Millipore, Bedford, MA, USA). Formic acid was acquired from Labkem (Barcelona, Spain). Murine macrophage cell line RAW 264.7 were purchased from the American Type Culture Collection (LGC Standards S.L.U., Barcelona, Spain). Dulbecco’s Modified Eagle Medium with GlutaMAX™ supplement (DMEM + GlutaMAX), fetal bovine serum (FBS) and penicillin-streptomycin solution were obtained from GIBCO, Invitrogen (Grand Island, NY, USA). 3-*O*-Caffeoylquinic acid and 5-*O*-caffeoylquinic acid were obtained from Biopurify Phytochemicals Ltd. (Chengdu, China). Catechin, epicatechin, vitexin, and isovitexin were purchased from Extrasynthese (Genay, France). Vicenin-2 was obtained from HWI Analytik GMBH (Rülzheim, Germany).

### 2.2. Plant Material

Leaves and stem bark of *F. curtipes* Corner were collected in Kasetsart University, Chatuchak, Bangkok, Thailand, in January 2018, specimens being identified by Prof. Dr. Srunya Vajrodaya (Department of Botany, Faculty of Science, Kasetsart University). A voucher specimen (PCERU_FC0005) was deposited at the Phyto-Chemodiversity and Ecology Research Unit, Faculty of Science, Kasetsart University.

### 2.3. Extraction

Leaves and stem barks were air-dried and ground to fine powder with a mean particle size ≤910 μm. A total of 51.1 and 71.8 g of leaves and stem bark powdered material, respectively, were macerated in 1 L of methanol for 7 days. The resulting extracts were filtered through Whatman^®^ grade 1 filtration paper (Sigma-Aldrich, St. Louis, MO, USA) using a Büchner funnel, and concentrated to dryness under reduced pressure in a Rotavapor^®^ R-210 (Büchi, Mumbai, India), yielding 4.8 and 4.0 g of extracts for leaves and stem bark, respectively. The yields of extraction with methanol from the leaves and stem bark were 9.4% and 5.6%, respectively.

### 2.4. Phenolic Profile Characterization

#### 2.4.1. HPLC-DAD-ESI (Ion Trap)/MS^n^ Qualitative Analysis

Phenolic profiles were obtained under the same chromatographic conditions reported by Ferreres et al. [12], with minor modifications on the gradient. Elution was performed with water-formic acid (1%) (A) and acetonitrile (B) as mobile phase, starting with 5% B and using a gradient to obtain 30% B at 20 min and 50% B at 30 min. The flow rate was 800 µL/min and the injection volume 20 µL. Chromatograms were recorded at 280 and 340 nm, and the full scan mass covered the range from *m*/*z* 100 up to 1500.

#### 2.4.2. UPLC-ESI-QTOF-MS Qualitative Analysis

Determination of the exact mass was carried out using an Agilent 1290 Infinity LC system coupled to the 6550 Accurate-Mass QTOF (Agilent Technologies, Waldbronn, Germany) with an electrospray interface (Jet Stream Technology). Samples (2 µL) were injected onto a reverse phase Kinetex column (1.7 µm, C18, 100 Å, 50 × 2.1 mm; Phenomenex, Macclesfield, UK) with a SecurityGuard ULTRA Cartridges of the same material operating at 30 °C and a flow rate of 0.5 mL min^−1^. The mobile phase used was a mixture of acidified water (0.1% formic acid) (A) and acidified acetonitrile (0.1% formic acid) (B). Compounds were separated using the following gradient conditions: 0 min 5% B, obtaining 30% B at 12 min and 50% B at 15 min. The optimal conditions for the electrospray interface were the following: gas temperature 280 °C, drying gas 11 L min^−1^, nebulizer pressure 45 psi, sheath gas temperature 400 °C, sheath gas flow 12 L min^−1^. The MS system was operated in negative ion mode with the mass range set at *m*/*z* 100–1500 in full scan resolution mode. Further conditions as described in Garcia et al. [13].

#### 2.4.3. HPLC-DAD Quantitative Analysis

The gradient and mobile phases were the same as above described. Dried methanol extracts obtained from the stem bark (150 mg/mL) and the leaves (70 mg/mL) of *F. curtipes* were dissolved in methanol and filtered through a 0.45 µM pore size membrane (Millipore, Bedford, USA) before injection (20 µL). Samples were analyzed in triplicate on an analytical HPLC unit (Gilson Medical Electronics, Villers le Bel, France), using a RP-Kinetex C18 (150 × 4.6 mm, 5 µm particle size, 100 Å pore size) column (Phenomenex, USA). Detection was achieved with an Agilent 1260 series diode array detector (Agilent Technologies, Waldbronn, Germany). Spectral data were collected in the range of 200–700 nm, chromatograms being recorded at 280 and 340 nm. Data were processed on Clarity software system, version 5.04.158 (DataApex, Ltd., Prague, Czech Republic). 

An equation of linear regression obtained from the respective calibration curve (concentration vs. optical absorbance) was built with six concentrations of external standards (in triplicate). Flavan-3-ols (**2**, **5**–**7**, **9**, **12**, **13**, **15**, and **17**) and aviculin (**19**) were quantitated using calibration curves generated at 280 nm; caffeoylquinic acids (**1**, **3**, and **4**) and apigenin derivatives (**8**, **10**, **11**, **14**, **16**, and **18**) were quantitated using calibration curves generated at 340 nm. 3-*O*-Caffeoylquinic acid (**1**), catechin (**2**), 5-*O*-caffeoylquinic acid (**4**), epicatechin (**7**), vicenin-2 (**8**), vitexin (**16**) and isovitexin (**18**) were quantitated as themselves, while the remaining compounds were quantitated against the standard curve of a structurally analogous compound: **3** was quantitated as 5-*O*-caffeoylquinic acid, **5**, **9**, and **17** as epicatechin, **6** as catechin, **10**, **11**, and **14** as vicenin-2 and **12**, **13**, **15**, **19**, **20**, and **21** as epigallocatechin. Linearity was determined from the coefficients of determination (*R*^2^) of the calibration curves. The limit of detection (LOD) and the limit of quantification (LOQ) were calculated from the residual standard deviation (*σ*) of the regression curves and the slopes (*S*), according to the following equations: LOD = 3.3*σ*/*S* and LOQ = 10*σ*/*S*.

### 2.5. 5-LOX Inhibition

5-LOX inhibition was evaluated by following the linoleic acid oxidation at 234 nm, according to a previously reported procedure [14]. Equal volumes of extract solution (20 µL) and 5-LOX (EC 1.13.11.12) from *Glycine max* (100 U/mL) were pre-incubated with 200 µL of phosphate buffer (0.1 M, pH 9), at room temperature for 5 min, on a 96 well-plate. The reaction was started by the addition of 20 µL of linolenic acid (4.18 mM in ethanol) and followed for 3 min at 234 nm (Multiskan ASCENT, Massachusetts, MA, USA). Results correspond to the mean ± SEM of three independent assays, each performed in triplicate. Quercetin was used as positive control.

### 2.6. RAW 264.7 Macrophages

#### 2.6.1. Cell Culture

Murine macrophage cell line RAW 264.7 (passages 54–71) were cultured in DMEM + GlutaMAX medium supplemented with 10% FBS and 1% penicillin/streptomycin, being maintained at 37 °C in a humidified atmosphere (5% CO_2_). After reaching 80%–90% confluence, cells were washed with fresh medium, scraped and subcultured on 96well plates for cell viability and NO levels assessment, as described below.

#### 2.6.2. Cell Viability

Cell viability was evaluated by monitoring the cell mitochondrial activity, using the MTT reduction assay, as previously reported [15]. Briefly, cells were cultured in 96-well plates (25,000 cells/well) and allowed to attach for 24 h. Posteriorly, cells were incubated with increasing concentrations of the plant extracts (62.5–1000 µg/mL) for 24 h. After, the medium was removed and MTT (final concentration 0.5 mg/mL) was added to each well, cells being incubated for 90 min at 37 °C. Formazan crystals were dissolved in a mixture of DMSO and isopropanol (3:1) and spectrophotometrically quantified at 560 nm in a microplate reader (Multiskan ASCENT, Massachusetts, MA, USA). Cell viability results were expressed as percentage of the control (untreated cells), corresponding to the mean ± SEM of three independent experiments, performed in triplicate.

#### 2.6.3. Determination of NO Levels in Culture Medium

Interference with NO levels in murine macrophage cell line RAW 264.7 was performed according to Ferreres et al. [15]. Cells were cultured in 96-well plates (35,000 cells/well), allowed to attach for 24 h and exposed to increasing concentrations of extract. After 2 h, 1 µg/mL (final concentration) of LPS was added to each well and plates were incubated for further 22 h at 37 °C, in a humidified atmosphere of 5% CO_2_. The nitrite formed by the conversion of NO in the culture medium was spectrophotometrically quantified at 540 nm in a microplate reader (Multiskan ASCENT, Massachusetts, MA, USA). NO levels were expressed as percentage of the NO in cells exposed to LPS (positive control) and correspond to the mean ± SEM of four independent experiments, performed in triplicate.

#### 2.6.4. Determination of l-Citrulline Levels in Culture Medium

Modulation of iNOS was determined by following the formation of l-arginine oxidative deamination bioproducts, NO and l-citrulline. Cells were cultured in 48-well plates (90,000 cells/well) and allowed to attach for 24 h. The medium was removed, and plates were incubated with medium containing increasing concentrations of the plant extracts. On the following 2 h, 1 µg/mL LPS was added to each well, plates being incubated for 22 h at 37 °C, in a humidified atmosphere of 5% CO_2_. After this period, the medium was removed and the iNOS substrate, l-arginine (50 µM) prepared in HBSS, was added to each well. The reaction was allowed to occur for 2 h, l-citrulline and NO levels being determined on the cell supernatant. NO levels were determined as described in Section 2.6.3., and l-citrulline was quantified as previously reported in [16]. Briefly, 250 µL of the culture supernatant was added to a mixture containing 79 mM diacetyl monoxime, 47.8 mM antipyrine E and 7.5 M H_2_SO_4_ and incubated at 96 °C for 25 min. After, the solution was cooled down to room temperature and the absorbance was read at 405 nm. l-citrulline levels were expressed as percentage of the levels in cells exposed to LPS (positive control) and correspond to the mean ± SEM of five independent experiments, performed in duplicate.

### 2.7. Determination of ^•^NO Levels in Non-Cellular System

Determination of ^•^NO levels in non-cellular system was evaluated through the Griess diazotization reaction as in Ferreres et al. [14]. Equal volumes of SNP (100 µL) and plant extract, prepared in KH_2_PO_4_ buffer (pH 7.4), were incubated for 1 h, under light at room temperature. Afterwards, the same volume of Griess reagent was added to each well and the plate was incubated for 10 min in the dark. Absorbance was read at 560 nm (Multiskan ASCENT, Massachusetts, MA, USA). ^•^NO scavenging activity was determined through the comparison of the optic density between the extracts and the control (buffer instead of sample) and correspond to the mean ± SEM of three independent experiments, performed in triplicate. Quercetin was used as positive control.

### 2.8. Statistical Analysis

All biological data were analyzed using Graph Pad Prism 6 software (San Diego, USA). Grubbs’ test was performed to detect significant outliers, differences at *p* < 0.05 being considered significant. On cellular assays, D’Agostino-Pearson normality test was performed and one-way analysis of variance (ANOVA) with Dunnett as post hoc test was used to compare significant differences amongst samples and controls (no extract treatment), *p* values lower than 0.05 being considered statistically significant.

## 3. Results

### 3.1. Characterization of the Phenolic Profile

#### 3.1.1. HPLC-DAD-ESI (Ion Trap)/MS*^n^* Qualitative Analysis

**Figure 1:**
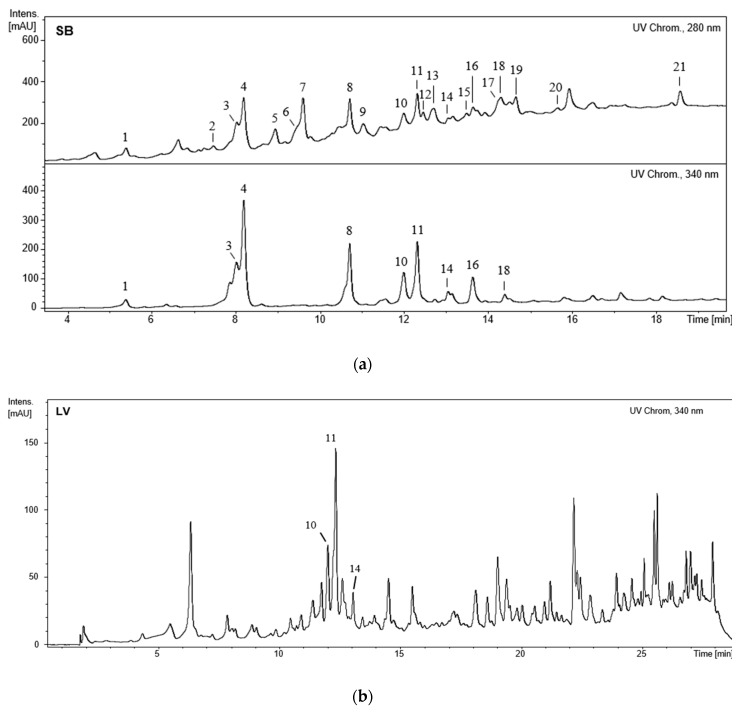
(**a**) HPLC-UV (280 and 340 nm) chromatogram of the methanol extract obtained from the stem bark (**SB**) of *Ficus curtipes*; (**b**) HPLC-UV (340 nm) chromatogram of the methanol extract obtained from the leaves (**LV**) of *F. curtipes*.

HPLC-DAD-ESI/MS*^n^* analysis of the methanol extracts obtained from the leaves and stem bark of *F. curtipes* allowed the identification of three and 21 compounds in each extract, respectively (Figure 1).

Compounds **1**, **3**, and **4** exhibited the same UV spectrum (300 sh, 326 nm) and the same deprotonated molecular ion (at *m*/*z* 353.0878), with molecular formula (C_16_H_18_O_9_) corresponding to caffeoylquinic acids. MS fragmentation of these compounds revealed a base peak at *m*/*z* 191; nevertheless, while in compounds **3** and **4** the ion at *m*/*z* 179 was weak or undetectable, in compound **1** fragmentation this ion had a high relative abundance (50%). Thus, according to Clifford et al. [17], compound **3** can be labeled as a chlorogenic acid isomer, while **1** and **4**, with the same chromatographic behavior of authentic standards, can unequivocally be identified as 3-*O*- caffeoylquinic acid and 5-*O*-caffeoylquinic acid, respectively. 

Compounds **8**, **10**, **11**, **14**, **16**, and **18** (Figure 1) exhibit an UV spectrum (272, 336 nm) of apigenin derivatives with substitutions at 6 and/or 8 positions, their MS pointing to *C-*glycosyl derivatives from apigenin.

Compounds **16** and **18** exhibit a deprotonated molecular ion at *m*/*z* 431.0977. In their MS fragmentations are observed ions at *m*/*z* 341 (aglycone + 71) and 311 (aglycone + 41) (Table 1), which, for mono-*C-*glycosylflavones, characterize the nature of the aglycone as apigenin [18]. As so, these compounds are apigenin-6/8-*C*-hexosides. The higher relative abundance of the ion at *m*/*z* 341 in compound **18** fragmentation comparing to that found for compound **16** (30% vs. 3%), the higher RP mobility of compound **16** (Table 1), and the occurrence of the [(M − H)-18]^−^ ion in **18** and its absence in **16** (data not shown in Table 1), indicate that the *C*-glycosylation in compound **16** occurs at position 8, while in **18** it is at position 6. Thus, considering the hexose to be glucose, compound **16** can be apigenin-8-*C*-glucoside (vitexin) and compound **18** apigenin-6-*C*-glucoside (isovitexin). Comparison with commercial standards allowed to unequivocally identify compounds **16** and **18** as vitexin and isovitexin, respectively.

The [M − H]^−^ of compound **10** at *m*/*z* 593.1506 indicates a diglycoside. In its MS fragmentation are observed the ions at *m*/*z* 341 and 311, which, as previously mentioned, are typical of apigenin-mono-*C*-glycosides. Ions at *m*/*z* 503 [(M − H)-90]^−^ and 473 [(M − H)-120]^−^, characteristic of MS fragmentation of a *C*-hexoside, the loss of a 162 amu fragment, and the absence of –180 (Table 1), indicating the presence of another hexose linked to a phenolic hydroxyl [19], are also detected. Consequently, compound **10** can correspond to an apigenin-*O*-hexoside-*C-*hexoside. While *O-*glycosylation generally occurs at position 7, *C-*glycosylation can appear either at position 6 or 8; thus, compound **10** can be labeled as apigenin-7-*O*-hexoside-6/8-*C-*hexoside corresponding, in the case of glucose being the hexose, to apigenin-7-*O*-glucoside-6/8-*C-*glucoside (isovitexin-7-*O*-glucoside or vitexin-7-*O*-glucoside). Unlike the previously described compounds, **10** was detected in both methanol extracts (Figure 1).

The fragmentation of compound **8**, with the same mass as **10**, does not exhibit the ions aglycone +71/+41, displaying instead the ions of aglycone +113/83 (*m*/*z* 383/353), typically found in di-*C*-glycosylflavones [18]. Ions from a *C*-hexoside fragmentation (–60/–90/–120) are also found, suggesting that compound **8** corresponds to apigenin-6,8-di-*C-*hexoside. Comparison with an authentic standard allowed to unambiguously identify **8** as apigenin-6,8-di-*C-*glucoside (vicenin-2).

Showing the same ions at *m*/*z* 383/353, as in **8,** and having the deprotonated molecular ion at *m*/*z* ~563.1400, compounds **11** and **14** correspond to apigenin-di-*C-*glycosides with both hexose and pentose on the sugar moiety. For both compounds, the relative abundance of the ion [(M − H)-60]^−^ is higher than the one found for compound **8**, due to the pentose; the higher abundance of this ion in compound **11** (15%), than in **14** (7%), indicates that the pentose is found at position 6 in compound **11**, as this is the preferential fragmentation position. On the other hand, the elution order of asymmetric di-*C*-glycosylflavones in RP indicates that the 6-*C*-pentosyl-8-*C*-hexosyl derivatives elute before the isomeric 6-*C*-hexosyl-8-*C*-pentosyl [18]. Thus, compound **11** can be labeled as apigenin-6-*C*-pentoside-8-*C*-hexoside and **14** as apigenin-6-*C*-hexoside-8-*C*-pentoside.

Not only **10**, but also compounds **14** and **11** were detected in the two methanol extracts, while the remaining compounds were solely identified on the extract obtained from the stem bark of *F. curtipes* (Figure 1B).

In the stem bark extract compounds structurally related with flavan-3-ols (**2**, **5**–**7**, **9**, **12**, **13**, **15**, **17**, **20**, and **21**) with characteristic UV spectrum (*λ*_max_ 280 nm) were also detected. Their deprotonated molecular ions indicate that these compounds must correspond to monomers, dimers, and trimers.

With [M − H]^−^ at *m*/*z* 289.0714 (C_15_H_14_O_6_), compounds **2**, **6**, and **7** have the same MS fragmentation pattern, characteristic of the most common natural monomers, catechin and epicatechin (Table 2). Comparison with authentic standards allowed the unequivocal identification of **2** and **7** as catechin and epicatechin, respectively.

Compounds **15**, **20**, and **21** (C_24_H_20_O_9_), displaying the same MS fragmentation, are substituted monomers coincident with cinchonain I type (a phenylpropanoid linked through a carbon-carbon linkage to the *C*-8 A-ring of epicatechin). Having the same deprotonated molecular ion (C_30_H_26_O_12_) and the same MS fragmentation, **5** and **17** correspond to catechin/epicatechin dimers, being known as procyanidin B (Table 2). Compounds **12** and **13** (C_39_H_32_O_15_) share the same MS fragmentation pattern, corresponding to cinchonain II type compounds, structurally related with procyanidin B but having a cinchonain I type similar substitution (a phenylpropanoid moiety on the *C*-8 position of the flavan superior unit). Furthermore, a catechin/epicatechin trimer (C_45_H_38_O_18_), procyanidin C type, was also detected (**9**). Compound (**19**) (*R*t: 14.6 min; UV: 282 nm; MS, [M − H]^−^: 505.2072, MS2: 359 (100%, −146)) exhibits an UV spectrum similar to those of the previous compounds and molecular formula C_26_H_34_O_10_. In its MS fragmentation a base peak at *m*/*z* 359 due to the loss of a rhamnosyl radical (146 amu fragment) is observed. This compound matches the structure of aviculin (isolariciresinol rhamnopyranoside), a rhamnoside lignan reported for the first time in *Polygonum aviculare* [20]. 

#### 3.1.2. HPLC-DAD Quantitative Analysis

HPLC-DAD quantitative analysis of the methanol extracts obtained from the stem bark and leaves was attainted using external calibration curves, allowing the quantitation of 17 flavonoids (**2**, **5**–**18**, **20**, **21**), three caffeoylquinic acid derivatives (**1**, **3**, **4**) and aviculin (**19**). Linear relationships with coefficient of determination (*R*^2^) above 0.998 were obtained for the external calibration curves built with the standard compounds (Table 3). 

The methanol extract obtained from the stem bark of the plant is predominantly characterized by the presence of cinchonain type I (**15**, **20**, and **21**) and type II (**12** and **13**) derivatives, corresponding to ca. 34% and 19% of the total quantifiable phenolic content, respectively (Table 4). As seen in Table 4, the cinchonain type I derivative **21** is the main component (1478.00 ± 18.67 mg/kg dry extract), the stem bark extract being also characterized by significant amounts of aviculin (**19**; 1024.17 ± 81.73 mg/kg dry extract). 

Despite the qualitative and quantitative dissimilarities between the extracts obtained from the two vegetal materials, the total amount of apigenin derivatives was similar (stem bark (**SB**): 618.67 ± 36.26 mg/kg dry extract; leaves (**LV**): 611.65 ± 29.81 mg/kg dry extract) (Table 4). While vicenin-2 (**8**) was the apigenin derivative identified in higher amounts in the stem bark extract, the main apigenin derivative, and simultaneously the major phenolic constituent present in the extract obtained from the leaves, was the apigenin-di-*C-*glycoside **11** (Table 4). 

### 3.2. 5-LOX Inhibition

**Figure 2:**
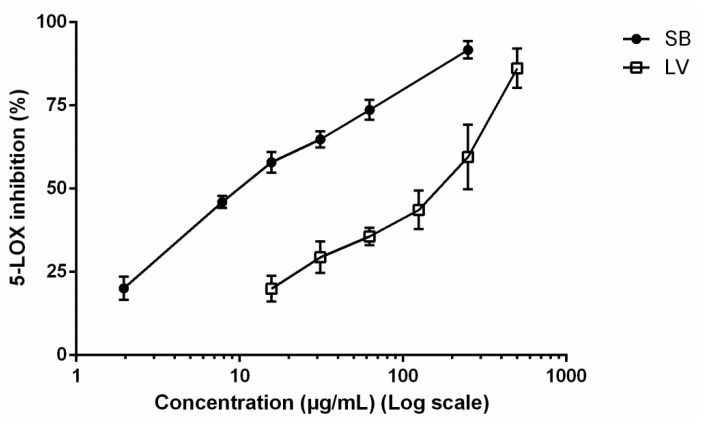
5-lipoxygenase (5-LOX) inhibition upon treatment with the methanol extracts obtained from the stem bark (**SB**) and leaves (**LV**) of *F. curtipes*. Data represent the mean ± SEM of three independent experiments, in triplicate.

As observed in Figure 2, both extracts were able to inhibit the activity of 5-LOX at the range of concentrations tested. The extract obtained from the stem bark proved to be more active, causing 91.72% ± 4.51% of inhibition at 250 µg/mL, while exposure to the leaf extract resulted in 59.57% ± 16.80% inhibition at the same concentration (Figure 2). Concentration-dependent inhibition allowed to determine the IC_50_ value for both extracts, 10.75 and 200.80 µg/mL being recorded upon treatment with the stem bark and leaf extract, respectively (Figure 2). While notably more potent than the leaf extract (Figure 2), the methanol extract obtained from the stem bark was 5-fold less effective than quercetin (positive control) with an IC_50_ value of 2.45 ± 0.65 µg/mL.

### 3.3. Effect on RAW 264.7 Macrophages 

**Figure 3:**
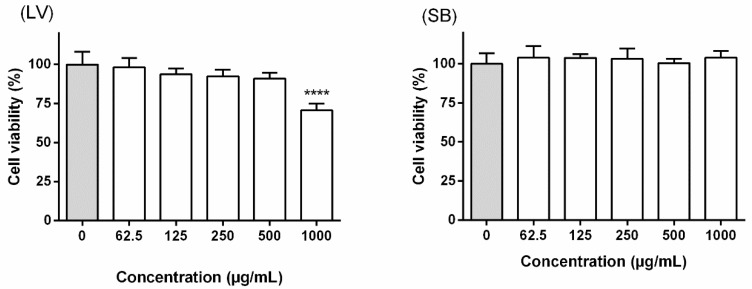
Effects of *F. curtipes* leaves (**LV**) and stem bark (**SB**) extracts on RAW 264.7 cells’ viability. Results represent the mean ± SEM of three independent experiments, performed in triplicate. **** *p* < 0.0001 compared to the respective control (no extract) (ANOVA, Tukey’s multiple comparison test).

In order to select working concentrations to evaluate the effects on NO levels, potential interference with the mitochondrial viability of RAW 264.7 macrophages was assessed through the MTT assay. While the extract obtained from the stem bark did not lead to cytotoxicity at concentrations ranging from 62.5 to 1000 µg/mL, a significant (*p* < 0.0001) reduction in the cell viability was observed upon treatment with the leaf extract at 1000 µg/mL (Figure 3). 

#### 3.3.1. Interference with NO Levels 

**Figure 4:**
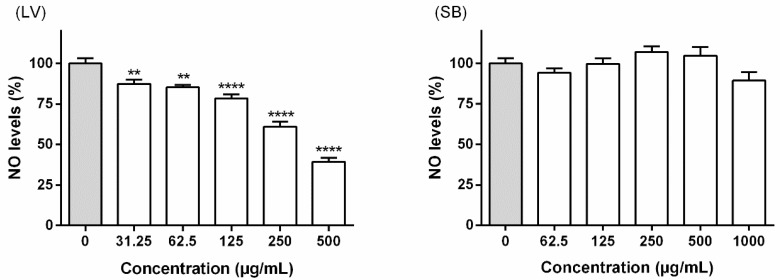
Effects of *F. curtipes* leaves (**LV**) and stem bark (**SB**) extracts on nitric oxide (NO) levels in RAW 264.7 macrophages. Cells were pre-treated for 2 h with the extracts, followed by 22 h co-treatment with lipopolysaccharide (LPS) (1 μg/mL). Results represent the mean ± SEM of four independent experiments, performed in triplicate. ** *p* < 0.01 and **** *p* < 0.0001 compared to the respective control (no extract added) (ANOVA, Tukey’s multiple comparison test).

Considering the potential cytotoxicity previously assessed, 1000 and 500 µg/mL were selected as the highest concentrations to evaluate, respectively, the effects of stem bark and leaf extracts on NO levels in LPS-challenged RAW 264.7 macrophages, measured as nitrite formation. After 22 h of concomitant exposure to LPS and extracts obtained from *F. curtipes*, no interference on the NO levels was observed with the stem bark extract (Figure 4). In contrast, the leaf extract was able to cause a significant concentration-dependent reduction on NO levels, at concentrations ranging from 31.25 to 500 µg/mL (Figure 4). It is worth also mentioning that at the highest tested concentration (500 µg/mL) the extract obtained from the leaves caused a significant (*p* < 0.0001) NO reduction (39.32% ± 8.69%), compared to the respective control (no extract treatment). 

#### 3.3.2. Interference with l-Citrulline Levels

**Figure 5:**
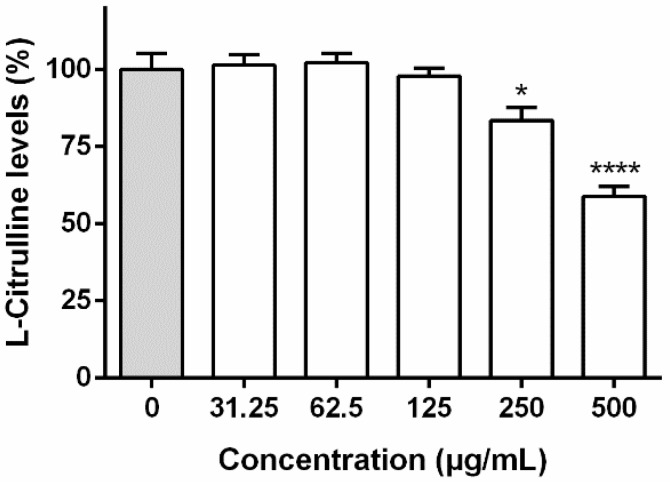
Interference with l-citrulline levels in LPS-stimulated RAW 264.7 macrophages upon 24 h exposure to the leaf extract obtained from *F. curtipes* (**LV**). Cells were pre-treated for 2 h with the extracts, followed by 22 h co-treatment with LPS (1 μg/mL). After, cells were incubated with 50 µM of l-arginine for 2 h. Results represent the mean ± SEM of six independent experiments, performed in duplicate. * *p* < 0.05 and **** *p* < 0.0001 compared to the respective control (no extract added) (ANOVA, Tukey’s multiple comparison test).

To assess the leaf extract interference with iNOS expression and/or its direct activity, l-citrulline levels were determined on LPS-stimulated RAW 264.7. As observed in Figure 5, there was a significant reduction in l-citrulline levels at concentrations higher than 125 µg/mL, a strong reduction (*p* < 0.0001) of ca. 58.90% ± 10.12% being observed at 500 µg/mL. 

### 3.4. Nitric Oxide Radical Levels in Non-Cellular System 

**Figure 6:**
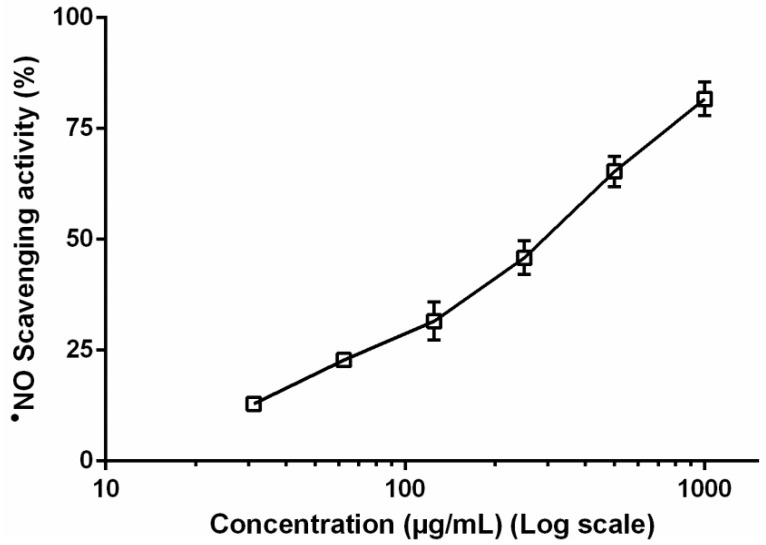
^●^NO scavenging capacity of the methanol extract obtained from the leaves of *F. curtipes* in the cell-free assay. Data represent the mean ± SEM of three independent experiments, performed in triplicate.

Considering the in vitro results obtained with RAW 264.7 cells, we found relevant to assess the ability of the methanol extract obtained from the leaves to scavenge the nitric oxide radical (^●^NO), produced through the photolytic decomposition of sodium nitroprusside. The leaf extract exhibited significant scavenging activity in a concentration-dependent manner (Figure 6), displaying an IC_50_ value of 304.8 µg/mL, ca. six times higher than the positive control (quercetin, IC_50_ = 54.42 µg/mL). At the range of concentrations used on the cellular assay (500–31.25 µg/mL) the scavenging activity varied between 12.92% ± 1.78% and 65.34% ± 5.92%.

## 4. Discussion

### 4.1. Phenolic Profile Characterization

In contrast with widely investigated *Ficus* species, such as *F. carica*, *F. racemosa*, or *F. religiosa*, there are no available records on the chemical profile of *F. curtipes*. The stem bark extract of this species is characterized by the occurrence of caffeoylquinic acids (**1**, **3**, and **4**), apigenin derivatives (**8**, **10**, **11**, **14**, **16**, and **18**), and predominantly flavan-3-ols (**2**, **5**–**7**, **9**, **12**, **13**, **15**, **17**, **20**, and **21**), while only apigenin derivatives (**10**, **11**, and **14**) were detected on the extract obtained from the leaves (Figure 1). As seen in Table 4, the main structural group of components occurring in the stem bark are phenylpropanoid-substituted monomeric (**15**, **20**, and **21**) and dimeric (**12** and **13**) flavan-3-ols, high amounts of procyanidins (**5**, **9**, and **17**) being also quantitated. Analogously, previous studies demonstrate that the occurrence of monomeric and condensed flavan-3-ols is common in *Ficus* spp., namely in external tissues; Konai et al. [21] reported that an aqueous extract obtained from *Ficus sycomorus* bark was also characterized by the occurrence of condensed tannins, particularly procyanidin type with 2,3-*cis* stereochemistry; the majority of the compounds identified in a methanol extract obtained from the stem bark of *Ficus microcarpa* correspond to catechin and epicatechin compounds, procyanidin B1 and B3 being also detected [22].

Additionally, caffeic acid derivatives, namely 3- (**1**) and 5-*O*-caffeoylquinic (**4**) acids, appear to be common metabolic products of the genus and frequently identified both in the leaves [23,24,25] and stem bark [25,26] of *Ficus* spp. Similarly, we have recently identified a series of mono-*C*-glycosides, such as vitexin (**16**) and isovitexin (**18**), and di-*C*-glycosides in aqueous extracts obtained from the leaves and stem bark of *F. exasperata* [25], additional studies being available on their occurrence in other *Ficus* spp. [23,27]. 

Curiously, while flavan-3-ols and apigenin derivatives are commonly reported in the genus, there was no evidence on the occurrence of the lignan glycoside aviculin (**19**) until the current study, herein identified as one of the main constituents of the methanol extract obtained from the stem bark of *F. curtipes* (Table 4). 

Quantitative profiles of the two extracts were found to be distinct, total quantifiable phenolic content of the extract obtained from the stem bark being approximately nine times higher than the leaves (5374.15 ± 436.61 vs. 611.65 ± 29.81 mg/kg dry extract) (Table 4). It is relevant to emphasize that, despite its lower phenolic content, the extract obtained from the leaves presented a higher concentration of the apigenin derivatives **10**, **11**, and **14**, the apigenin-6-*C*-hexoside-8-*C*-pentoside (**14**) content being ca. eight times higher on this extract. On the other hand, considering the total quantifiable phenolic content, the great abundance of cinchonain type compounds in the stem bark extract points this vegetal material of *F. curtipes* as a great source flavan-3-ols. 

### 4.2. 5-LOX Inhibition

The enzyme 5-LOX is involved in the first steps of the conversion of arachidonic acid into leukotrienes, which are primarily responsible for the initiation of the inflammatory process [23]. Besides their chemokinetic and chemotactic responses, leukotrienes also play a role in the recruitment, migration, adhesion, and degranulation of granulocyte cells, acting as potent modulators of the inflammatory process [28]. In fact, leukotrienes are intimately related with the pathophysiology of diseases, such as atherosclerosis, asthma, arthritis, and skin disorders with an inflammatory background, and became the focal point of many therapeutic approaches for the treatment of these conditions [28,29]. Hence, there is a rationale on the inhibition of 5-LOX as a proper therapeutic strategy for these leukotriene-related disorders management [29]. 

Our results demonstrate that the two extracts obtained from *F. curtipes* materials were able to inhibit 5-LOX in a concentration-dependent manner (Figure 2), the extract obtained from the stem bark being markedly more effective (IC_50_ = 10.75 µg/mL). At a first glance, it is tempting to relate the stronger inhibitory effects observed upon exposure to the stem bark extract to the higher amounts of phenolic constituents identified in the extract (5374.15 ± 436.61 mg/kg dry extract) in comparison with the leaf extract (611.5 ± 29.81 mg/kg dry extract) (Table 4). In fact, while phenolic compounds are generally described as prominent plant-derived inhibitors of 5-LOX product synthesis [28,30], individual constituents identified in stem bark extract can directly contribute to the observed inhibitory effects. Specifically concerning monomeric flavan-3-ols, it is worth referring to the effects upon 5-LOX dependent leukotriene C_4_ (LTC_4_) production in bone marrow-derived mast cells upon treatment with catechin (**2**) (20 µM), mediating 99.8% inhibition [31], being also reported to inhibit potato 5-LOX activity (IC_50_ value of 16.5 µg/mL) [32]. Relevantly, catechin (**2**) happens to be one of the main active compounds found in flavocoxid, an FDA-approved medical food for the treatment of osteoarthritis [33]. Additionally, epicatechin (**7**) has been reported to inhibit both human 5-LOX and rabbit 15-LOX, with IC_50_ values of 22 and 60 µM, correspondingly [34]. Such findings are in agreement with the reported decay of plasmatic pro-inflammatory cysteinyl leukotrienes observed after human intake of flavonoid-rich chocolate with substantial amounts of epicatechin (**7**) and related oligomers (procyanidins) [35]. Interestingly, Schewe and colleagues demonstrated that epicatechin (**7**) and its lower-molecular procyanidins inhibit both steps of 5-LOX catalysis: the deoxygenation of arachidonic acid to 5-hydroperoxy-6*E*,8*Z*,11*Z*,14*Z*-eicosatetraenoic acid and the following conversion to 5,6-leukotriene A_4_ [34]. Additionally, the potential contribution of the caffeoylquinic acids, constituting ca. 5% of the stem bark phenolic content (Table 4), should be taken into account; as recently reported by Gawlik-dziki et al. [36], 5-*O*-caffeoylquinic acid (**4**) acts as a potent soybean 5-LOX inhibitor, with an IC_50_ of 18.56 µg/mL. It is also worth mentioning the possible interference of the identified apigenin derivatives on the stem bark extract that were not detected in the leaves. While there are no available studies on the inhibitory capacity of vicenin-2 (**8**), both vitexin (**16**) and isovitexin (**18**) have already been reported as soybean 5-LOX [37] and 15-LOX inhibitors [38], with IC_50_ values of 5.1 and 107.1 µM, respectively. Hence, it is conceivable to assume the observed inhibitory effects towards 5-LOX to be related, at least partially, with the identified phenolic compounds.

### 4.3. Effect on RAW 264.7 Macrophages and ^●^NO Scavenging Capacity

The innate immunity system responds to the presence of pathogens or tissue injury through immune cells recruitment, namely monocytes and macrophages [39]. These cells are commonly responsible for the development of inflammatory-derived symptoms, namely edema, heat, redness, and pain [39]. When triggered by microbial endotoxins and/or endogenous inflammatory cytokines, macrophages become activated and start to express genes involved in host defense, leading to the production of reactive nitrogen species and bioactive lipids derived from arachidonic acid, contributing to the inflammatory process [39].

Appropriate cell models to assess anti-inflammatory properties include LPS-challenged murine macrophage RAW 264.7 cells [14,16]. LPS-derived activation leads to the upregulation of pro-inflammatory cytokines, such as IL-1, IL-6, or TNF-α, as well as pro-inflammatory proteins, including iNOS [39]. This enzyme catalyzes the conversion of the substrates O_2_ and l-arginine to l-citrulline and NO, being the main responsible for their overproduction [39]. Increased NO production is actively involved in the inflammatory process, and can be either beneficial, acting as a bactericidal, or harmful, causing DNA damage and protein oxidation [40]. This cellular mediator is a well-acknowledged inflammatory biomarker, inhibition of its production being a widely validated approach for the amelioration of the inflammatory response [39,40].

In order to ensure that the potential reduction of NO levels in RAW 264.7 macrophages was not related with cytotoxicity, interference with mitochondrial viability was firstly evaluated through the MTT reduction assay. As seen in Figure 3, solely the extract obtained from the leaves of *F. curtipes* caused a significant decrease on the cell viability of RAW 264.7 cells, a range of working concentrations between 31.25 and 500 µg/mL being consequently selected. The 24 h-exposure to the leaf extract led to a decrease on NO levels at all tested concentrations, a reduction to 39.32% ± 8.69% being recorded at 500 µg/mL (Figure 4). No effects were observed upon treatment with the extract obtained from the stem bark (Figure 4).

Since cellular NO levels decrement results from the interference with the iNOS expression and/or activity, and/or the direct scavenging of this reactive species, additional in vitro assays were geared as an attempt to explain the observed effects upon treatment with the leaf extract. This extract was further evaluated for its ability to reduce l-citrulline levels on the cells’ supernatant (Figure 5) and to scavenge ^•^NO in a non-cellular system (Figure 6). Results evidence that, at 250 and 500 µg/mL, the extract acts through a bimodal mechanism, directly interfering with iNOS activity and also displaying a significant scavenging effect towards ^•^NO, thus explaining the reduction in NO levels in LPS-activated RAW 264.7 macrophages (Figure 4). However, the effects observed at lower concentrations (Figure 4) are likely to be predominantly related with a radical scavenging effect (Figure 6).

It is plausible to consider that the di-*C*-glycosides **10**, **11**, and **14** contribute to the direct modulation of iNOS (Figure 5), as apigenin derivatives are known to interfere with the enzyme [41,42]. SAR studies with a series of flavonoids allowed to ascribe the structural features resulting in iNOS downregulation in RAW 264.7 macrophages, such as the importance of the *C*-2,3 unsaturation, the A-ring di-hydroxy *meta*-substitution and the 4’-hydroxyl group, flavones being within the optimal structures, if not the best [43,44]. Additionally, the strong antiradical effects observed upon treatment with *F. curtipes* leaf extract may be partially mediated by the presence of the apigenin di-*C*-glycosides (**10**, **11**, and **14**), as flavones have long been identified as efficient ^•^NO scavengers [45]. In fact, apigenin derivatives follow the optimal structural requirements, namely the presence of hydroxyl substituents in *C*5 and *C*4′, as well as the C-ring carbonyl function [46].

Overall, our data prove that the extract obtained from the leaves of *F. curtipes* significantly reduced NO levels in RAW 264.7 macrophages, acting through the combination of radical scavenging properties and iNOS modulation, which can thereby result from direct enzyme inhibition and/or enzyme expression inhibition. The results recorded here are reported for the first time in this species, providing initial proof on its effect upon a well-recognized inflammatory pathway and stimulating further molecular approaches to thoroughly comprehend the conceivable anti-inflammatory potential of this plant material.

## 5. Conclusions

Biological data generated here provides preliminary evidence on the conceivable modulatory effect of *F. curtipes* on inflammatory-related conditions. While the extract obtained from the stem bark proved to efficiently inhibit 5-LOX activity, the leaf extract was found to significantly interfere with NO levels in LPS-challenged RAW 264.7 macrophages, as a consequence of a significant anti-radical effect and modulation of iNOS expression and/or activity.

Furthermore, the species is also valorized as a source of a series of structurally diverse phenolic compounds. The phenolic profile of the species is disclosed for the first time, evidencing a chemical richness characterized by the occurrence of apigenin, as well as flavan-3-ols, some of which are well known for their anti-inflammatory ability and therapeutic utility.

Taking the above into account, the present work provides further insights not only on *F. curtipes* polyphenolic composition, but also on its biological properties, encouraging further molecular approaches in order to thoroughly comprehend the anti-inflammatory potential of this plant species.

## Figures and Tables

**Table 1:** *R*t, molecular formula, and MS [M − H]^−^ and MS^2^[M − H]^−^ data for the apigenin derivatives detected in the methanol extract obtained from the stem bark of *F. curtipes*. ^1^

Compounds	*R*t (min)	Formula (M)	[M − H]^−^ *m*/*z*	MS^3^[M − H]^−^, *m*/*z* (%)							
				−60	−90	−120	−162	Agl^2^ + 113	Agl^2^ + 83	Agl^2^ + 71	Agl^2^ + 41
**8**	10.7	C_27_H_30_O_15_	593.1508	533 (2)	503 (30)	473 (100)		383 (45)	353 (80)		
**10**	12.0	C_27_H_30_O_15_	593.1506		503 (2)	473 (30)	431 (60)			341 (15)	311 (100)
**11**	12.3	C_26_H_28_O_14_	563.1396	503 (15)	473 (80)	443 (100)		383 (65)	353 (90)		
**14**	13.0	C_26_H_28_O_14_	563.1402	503 (7)	473 (85)	443 (50)		383 (75)	353 (100)		
**16**	13.6	C_21_H_20_O_10_	431.0980							341 (3)	311 (100)
**18**	14.3	C_21_H_20_O_10_	431.0974							341 (30)	311 (100)

^1^ Main observed fragments. ^2^ Agl: aglycone.

**Table 2:** *R*t, molecular formula, and MS [M − H]^−^ and MS^2^[M − H]^−^ data for the flavan-3-ols derivatives detected in the methanol extract of the stem bark from *F. curtipes*.

Compounds	*R*t (min)	Formula (M)	[M − H]^−^ *m*/*z*	MS^3^[M − H]^−^ *m*/*z* (%)
**2**	7.4	C_15_H_14_O_6_	289.0717	245(100), 205(50)
**5**	8.9	C_30_H_26_O_12_	577.1345	425(100), 407(80), 289(25)
**6**	9.4	C_15_H_14_O_6_	289.0714	245(100), 205(35)
**7**	9.6	C_15_H_14_O_6_	289.0714	245(100), 205(30)
**9**	11.0	C_45_H_38_O_18_	865.1988	695(100), 577(90), 425(60), 407(60), 287(30)
**12**	12.4	C_39_H_32_O_15_	739.1651	587(100), 569(25), 435(65), 417(40), 339(30), 289(20)
**13**	12.6	C_39_H_32_O_15_	739.1678	587(100), 569(30), 435(50), 417(10), 339(30), 289(15)
**15**	13.4	C_24_H_20_O_9_	451.1020	341(10)
**17**	14.3	C_30_H_26_O_12_	577.1347	425(100), 407(70), 289(30)
**20**	15.6	C_24_H_20_O_9_	451.1026	341(100)
**21**	18.5	C_24_H_20_O_9_	451.1024	341(10)

**Table 3:** Linear regression equation analysis, limit of detection (LOD) ^a^ and limit of quantification (LOQ) ^b^, for external standards.

Standard	Regression Equation			Linearity Range (µg/mL)	LOD (µg/mL)	LOQ (µg/mL)
	Slope (*σ*)	Intercept (*b*)	*R*^2^ (n = 3)			
3-*O*-Caffeoylquinic acid	44.661	−17.577	0.999	2–32	0.439	1.330
Catechin	16.114	22.846	0.998	141–4.4	1.040	3.152
5-*O*-Caffeoylquinic acid	157.450	100.020	0,999	7.5–120	2.125	6.439
Epicatechin	35.932	44.752	0.998	145–4.5	0.691	2.094
Vicenin-2	55.527	−0.958	0.999	3–48	0.606	1.837
Epigallocatechin	3.291	−5.585	0.999	250–3.9	1.125	3.408
Vitexin	80.631	73.566	0.997	3–48	0.256	0.777
Isovitexin	98.771	27.029	0.998	1–16	0.013	0.040

^a^ Limit of detection, ^b^ limit of quantification.

**Table 4:** Content of phenolic compounds on the methanol extracts obtained from the stem bark (**SB**) and leaves (**LV**) of *F. curtipes* (mg/kg dry extract). ^a^

Compound		SB	LV
**1**	3-*O*-Caffeoylquinic acid	33.79 ± 2.45	Nd
**2**	Catechin	10.17 ± 2.64	Nd
**3**	Chlorogenic acid isomer	33.78 ± 2.29	Nd
**4**	5-*O*-Caffeoylquinic acid	201.23 ± 6.88	Nd
**5**	Procyanidin type B	52.17 ± 1.10	Nd
**6**	Catechin/Epicatechin derivative	129.38 ± 19.16	Nd
**7**	Epicatechin	377.51 ± 21.29	Nd
**8**	Vicenin-2	218.23 ± 11.41	Nd
**9**	Procyanidin type C	27.44 ± 0.82	Nd
**10**	Apigenin-7-*O*-Hex-6/8-*C*-Hex	96.99 ± 6.59	152.17 ± 2.83
**11**	Apigenin-6-*C*-Pt-8-*C*-Hex	204.19 ± 9.89	381.20 ± 10.58
**12**	Cinchonain type II	280.94 ± 56.52	Nd
**13**	Cinchonain type II	727.65 ± 67.62	Nd
**14**	Apigenin-6-*C*-Hex-8-*C*-Pent	8.46 ± 1.13	78.27 ± 16.40
**15**	Cinchonain type I	293.45 ± 113.90	Nd
**16**	Vitexin	72.73 ± 4.29	Nd
**17**	Procyanidin type B	8.81 ± 1.69	Nd
**18**	Isovitexin	18.08 ± 2.96	Nd
**19**	Aviculin	1024.17 ± 81.73	Nd
**20**	Cinchonain type I	77.30 ± 11.35	Nd
**21**	Cinchonain type I	1478.00 ± 18.67	Nd
	**Total**	**5374.15 ± 436.61**	**611.5 ± 29.81**

^a^ Results correspond to mean ± SD (*n* = 3); Nd (Not detected). Hex: hexoside; Pent: pentoside.
